# A computational platform to maintain and migrate manual functional annotations for BioCyc databases

**DOI:** 10.1186/s12918-014-0115-1

**Published:** 2014-10-12

**Authors:** Jesse R Walsh, Taner Z Sen, Julie A Dickerson

**Affiliations:** Bioinformatics and Computational Biology Program, Iowa State University, Ames, IA USA; Electrical and Computer Engineering Department, Iowa State University, Ames, IA USA; USDA-ARS Corn Insects and Crop Genetics Research Unit, Iowa State University, Ames, IA USA; Department of Genetics, Development and Cell Biology, Iowa State University, Ames, IA USA

**Keywords:** Annotation tool, BioCyc, Pathway/Genome database, JavaCycO

## Abstract

**Background:**

BioCyc databases are an important resource for information on biological pathways and genomic data. Such databases represent the accumulation of biological data, some of which has been manually curated from literature. An essential feature of these databases is the continuing data integration as new knowledge is discovered. As functional annotations are improved, scalable methods are needed for curators to manage annotations without detailed knowledge of the specific design of the BioCyc database.

**Results:**

We have developed CycTools, a software tool which allows curators to maintain functional annotations in a model organism database. This tool builds on existing software to improve and simplify annotation data imports of user provided data into BioCyc databases. Additionally, CycTools automatically resolves synonyms and alternate identifiers contained within the database into the appropriate internal identifiers.

**Conclusions:**

Automating steps in the manual data entry process can improve curation efforts for major biological databases. The functionality of CycTools is demonstrated by transferring GO term annotations from MaizeCyc to matching proteins in CornCyc, both maize metabolic pathway databases available at MaizeGDB, and by creating strain specific databases for metabolic engineering.

**Electronic supplementary material:**

The online version of this article (doi:10.1186/s12918-014-0115-1) contains supplementary material, which is available to authorized users.

## Background

Lower costs in genomic sequencing and improved methods of generating computationally predicted functional annotations has led to the development of many model organism databases using the BioCyc framework [1]. While computationally derived draft model organism databases provide useful starting points for storing biological knowledge, computationally predicted annotations are known to suffer from significant false negative rates [2]. The accuracy of annotations can be substantially improved by providing manual annotations mined from literature by expert curators. Unfortunately, manual curation efforts have not kept up with the proliferation of new databases. There are currently over 3500 databases in the BioCyc collection, however only 42 of these currently receive moderate or intensive manual review [3].

Among the databases that receive manual review, maintaining manually curated data can present a challenge. When an improved reference sequence is released for an organism, the BioCyc database representing that organism must be recreated in order to incorporate the new sequence data. While computationally predicted annotations within the database should be updated using the new input data, it is usually preferred to keep existing manual annotations even if the computational annotations are more recent. There is a need for tools which can assist curators in persisting manually curated data through the update process either through automation or by providing pipelines for the transfer of manual annotations of these databases. Additionally, when several distinct databases host biological data for the same organism, it is desirable to share manually curated annotations between these databases in order to improve data accuracy without duplicating curator efforts. In order to facilitate the transfer of data between databases, robust import and export features must be made available.

**Figure 1:**
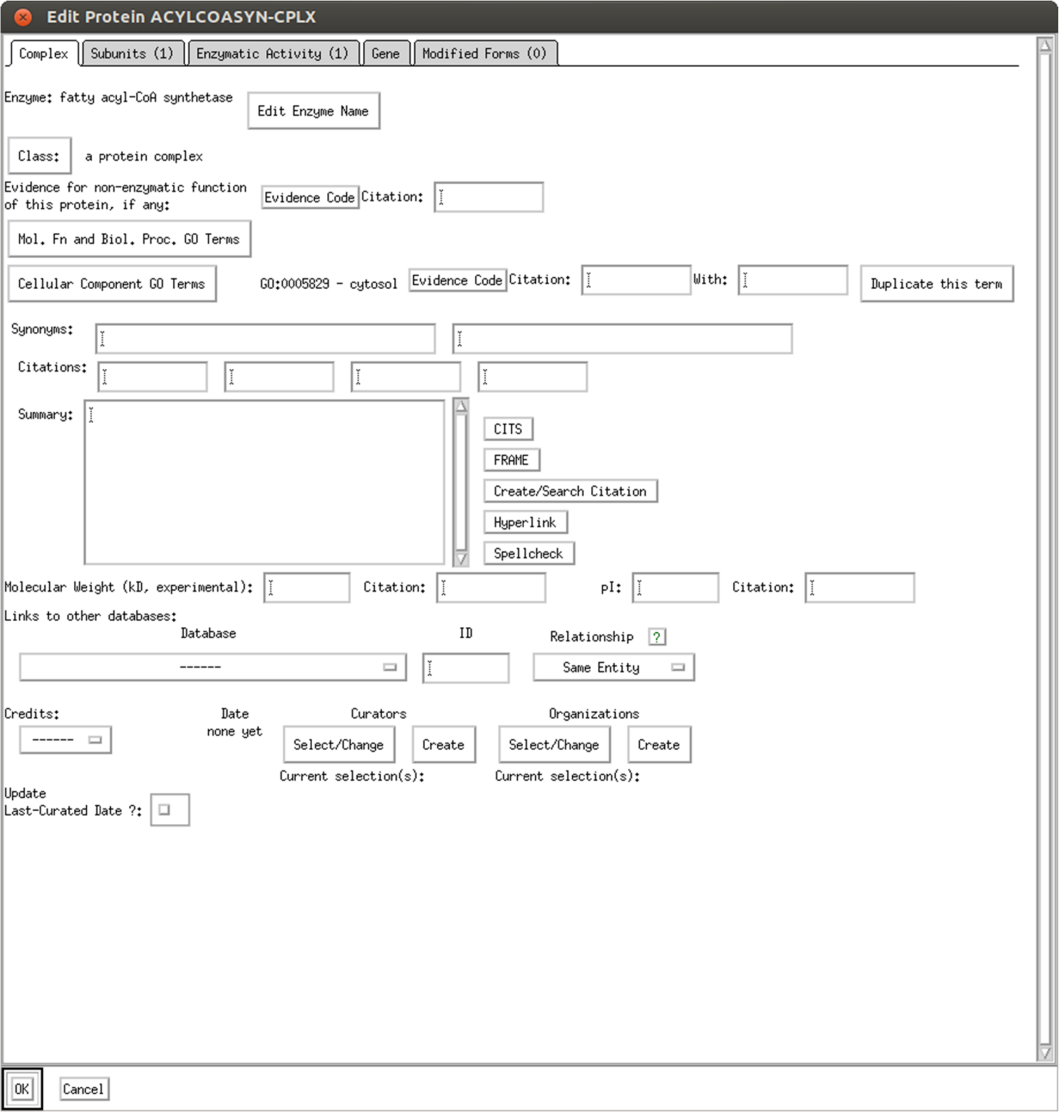
**Screenshot of pathway tools protein editor.** Editing database objects through the Pathway Tools software editors is done by entering information into forms which describe information specific to the type of object being edited.

Pathway Tools [4], the software which supports development and management of BioCyc databases, provides several options for updating a BioCyc database. Changes may be made manually within the pathway tools software by first locating the object to update and then entering edit mode to make the changes to that object, as shown in Figure 1. Each object type (protein, gene, metabolite, etc.) has a specific data entry form, which can be filled out and saved. While this method allows the curator to directly review and verify the changes entered into the database, it is inefficient when performing large numbers of updates.

Pathway Tools supports data imports through two file formats, “spreadsheet format” or “Lisp-format”. Examples are provided in Additional file 1. The spreadsheet format imports are limited in that some data cannot be imported using this method, including GO term annotations, stoichiometry, and cellular localization. While the Lisp-format supports the import of these data types, it requires users to have an understanding of the Lisp data structure implemented in the BioCyc framework and is not easily converted to other standard formats.

A final import option provided by Pathway Tools is through an application programming interface (API), which exposes low level access to the BioCyc data structure. The API is very flexible in that users can design queries to suit their specific needs, but they must have a detailed understanding of the internal structure of a BioCyc database in order to do so. Certain modifications to a BioCyc database, such as GO term annotations, require additional steps in order to maintain the referential integrity of the database. This provides further barriers to use, as users must have an understanding of how Pathway Tools implements storage of these features.

Despite the diversity of import methods provided by Pathway Tools, there remains a need for an import pipeline which is both capable of importing slot-value annotation data in batch and accessible to researchers who are not experts in programming or BioCyc database structure. CycTools is a graphical interface for the BioCyc family of databases which improves data management by providing methods which can import slot-value annotation data in batch.

## Implementation

### CycTools dependencies

**Figure 2:**
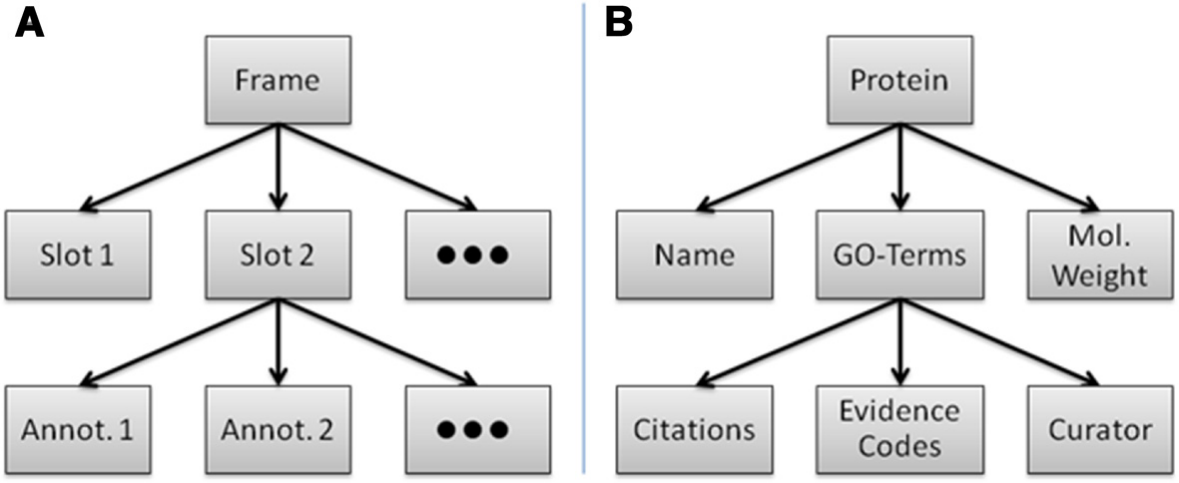
**Structure of Frames in a PGDB.** **A)** Frames describe objects in the database. Slots contain information about the frame object, and annotations contain meta-information about slot information. **B)** a protein is represented by a frame in the database. Examples of slots which describe the protein include the protein name, molecular weight, and GO-Term assignments. Annotations of the GO term information include citations, evidence codes, and information on the person who curated this GO term assignment.

BioCyc is a family of databases built using the BioCyc Framework. Each member database of the BioCyc collection typically represents the pathway and genomic data of a specific organism. BioCyc databases are built on the Frame Representation System (FRS) known as Ocelot [5], which extends the Generic Frame Protocol (GFP). The native storage format for BioCyc data is an object oriented database representation based on frames. The hierarchical nature of data represented in a frame can be seen in Figure 2. A frame is a high level container that groups information regarding either biological entities (genes, proteins, transcripts, compounds, etc.) or biological relationships (reactions, pathways, regulation, etc.). Information about the object a frame represents can be stored in either slots or slot-value-annotations. Information stored in slots describes the frame (i.e. the name of the object, its physical properties, or annotations assigned to it), while information in slot-value-annotations provides context for the information in the slots (i.e. pubmed citations, author credits, or experimental evidence codes). The data stored in frames and slots in the database can be accessed programmatically through the Pathway Tools API.

The API exposes many of the internal functions of Pathway Tools and allows low level access to the internal data structure of any BioCyc database hosted by Pathway Tools. Advanced users can create third-party software which can read or write to BioCyc databases using customized queries. The API is designed to support the Lisp programming language, but the libraries PerlCyc [6] and JavaCycO [7] allow users to access the API through Perl and Java respectively.

JavaCycO is an object-oriented improvement to the JavaCyc library. JavaCycO contains the JavaCyc [6] class and is fully backwards compatible with it. In addition to extending and improving the functionality of JavaCyc, JavaCycO provides a client-server model for accessing the Pathway Tools API. By running the server “JavaCycServer” on the same machine as Pathway Tools, JavaCycO provides remote access to the Pathway Tools API to JavaCycO clients. CycTools depends on the JavaCycO library to provide access to the Pathway Tools API in order to read and write to a BioCyc database. More details on installing these dependencies can be found in Additional file 2.

### Cloning a database

Generally speaking, CycTools can modify any BioCyc database hosted by Pathway Tools. Two notable exceptions to this are the MetaCyc and EcoCyc databases, which are integrated into Pathway Tools and flagged as read-only. Since these databases can not be removed or modified, the only way to edit them is to edit a copy. Pathway Tools will also refuse to load two databases with the same name, which prevents the user from simply installing a second copy of a database without first renaming and modifying several of the files and folders within the copy. This restriction will also prevent the user from creating and hosting several versions of a database in the same Pathway Tools instance. In order to circumvent this restriction, a bash script which automatically clones a database and modifies the appropriate files was created. This tool is made available in Additional file 3.

### Overview of import process

**Figure 3:**
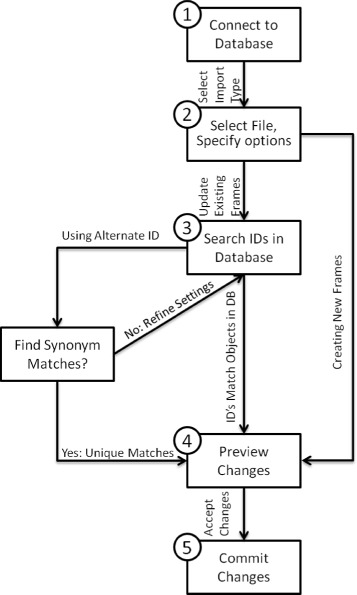
**Import process diagram.** Synonym based search automatically occurs if import file does not contain Frame IDs. Only unique matches to frame IDs are allowed in order to prevent ambiguity in the import process.

The CycTools import function provides a graphical pipeline for importing spreadsheet data into frame objects in the Pathway Genome Database (PGDB). The import utility takes as input a comma-separated data file, maps the data to frames in the PGDB, previews the resulting changes to the PGDB, and performs the update of the PGDB as shown in Figure 3.

**Figure 4:**
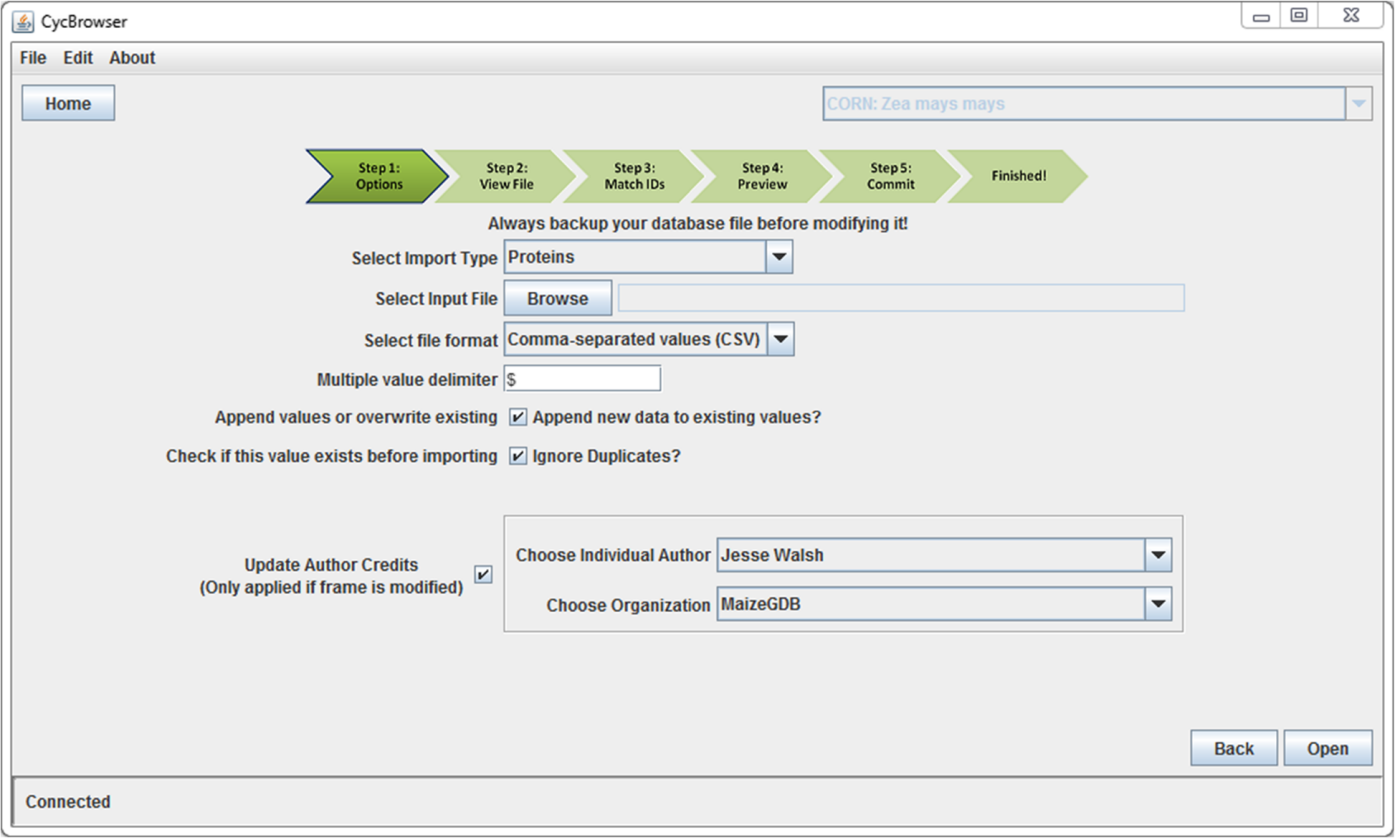
**CycTools import screen.** CycTools provides a multi-step process for importing user data. Several options are available for users to interact with existing data. Users can also specify an author or organization to assign credit for the revision of a database frame object.

CycTools must be able to connect to a server running Pathway Tools in API mode and JavaCycO. Once connected, the user will select one of the available import types: import slot data, import slot-value annotation data, import GO annotations, delete frames, or create transcriptional regulation frames. This determines the format of the import file and how the imported data are applied to database objects. Additional options are available which allow the user to specify how to handle existing data in a slot or annotation which will be modified during import, shown in Figure 4.

If the overwrite option is set, CycTools will first delete the existing data in a slot or annotation before writing the user provided data to that slot or annotation. If the ignore duplicates option is set, CycTools will check each new value against each existing values in a slot or annotation. If the new value exactly matches an existing value, it will not be added to the slot or annotation. This option will prevent the user from adding a duplicate value to a slot or annotation, but will not remove an existing duplication. Thus, if a protein were to be annotated with a single GO term twice, this option will prevent CycTools from adding a third identical annotation using that GO term, but would leave the existing annotations.

The author credits option allows the user to assign credit to an individual or organization for each frame updated during the import process. CycTools autofills a list of curators and organizations described in the currently selected database. For each frame updated during the import, the frame is modified to append the curator or organization to the “CREDITS” slot. This update is annotated as a revision to the frame and is timestamped to the current system time.

#### GO term annotations

GO term annotation imports are handled slightly different from other annotations imports. In particular, Pathway Tools has specific requirements for the storage of GO term descriptions within a BioCyc database. The Pathway Tools API provides a method called “import-go-terms” which automatically creates the necessary frames when provided with a valid GO term. Pathway Tools is packaged with a file containing GO term information which is used by this method to populate the GO term frames it creates. CycTools makes a call to “import-go-terms” once for each GO term that appears during a GO term annotation import.

#### Resolving alternate identifiers to database frames

Each frame object in the database is uniquely identified by an internal identifier known as the frame ID. The BioCyc framework supports annotating frames with alternate identifiers, such as those which are commonly used in literature to refer to genes, proteins, and other biological objects. For example, “PYRUVATE” in EcoCyc has the synonyms alpha-ketopropionic acid, BTS, *α*-ketopropionic acid, acetylformic acid, pyroracemic acid, 2-oxopropanoic acid, pyruvic acid, 2-oxopropanoate, and 2-oxo-propionic acid. Despite the availability of these alternate identifiers, all queries to the database must resolve to valid frame IDs. A key benefit of CycTools is support for automatically resolving alternate identifiers into frame IDs, removing the need for researchers to perform the conversion manually. Alternate identifiers must already be annotated to the object they identify within the database and must be stored in one of the slots designated as a “name” slot in Pathway Tools. These slots typically include the “accession” slot, “common-name” slot, “synonym” slot, and foreign database identifiers used in the “dblink” slot, but can vary with object type.

During the import process, CycTools attempts to resolve all user provided identifiers into frame IDs. First, CycTools checks if the user provided identifiers match exactly to any existing frame IDs. If all identifiers are determined to be valid frame IDs, no further action is needed and the ID resolution step is skipped. If one or more IDs are not valid frame IDs, CycTools will attempt to resolve them into valid frame IDs using an indexed text search within the database using the “substring-search” method provided by the Pathway Tools API. The substring-search command can find objects with frame IDs that exactly match the search string which match to a substring of any “name” slot. The search term provided by the user must be at least 3 characters with no commas or spaces. This method requires the user to specify the object type to search and the alternate identifiers to be converted to frame IDs. For each identifier in the import file, CycTools requires that the searched term match exactly and entirely to at least one synonym provided by the database for the matching object. Thus, while substring search will match a partial identifier to a frame, CycTools enforces a stricter matching policy by filtering out matches that do not contain complete matches to an alternate identifier. Additionally, CycTools requires that only one such matching object be found in the database. If the search returns only a single frame, that frame’s ID is substituted for the searched term. If multiple matches or no match is found, the user is given the option to ignore that data during import, or to cancel the import process altogether.

### Create transcriptional regulation frames

Importing novel transcriptional regulatory interactions requires creating regulation frames within the BioCyc database to represent the interaction. Since this import type generates new frames rather than modifying existing ones, the user does not provide frame identifiers with the import data. As a result, no frame ID search is necessary. CycTools instead requests unique sequential identifiers for each new regulation object created. CycTools is not able to recognize if an equivalent regulatory interaction exists in another regulation frame, and therefore relies on the user to ensure that regulatory interactions are not duplicated.

### Delete frames

CycTools implements frame deletion using the Pathway Tools API method “delete-frame-and-dependents”. This method detects the object type of the frame which is being deleted and attempts to also delete any frames which depend on the deleted frame. For example, deleting a gene frame will also delete the gene’s products, and potentially enzymatic reactions which depend on an enzyme produced by the gene. Regulation frames and history note frames linked to the deleted frame are also deleted.

#### Preview changes

Before any permanent modification is made to the database, the user can preview the pending changes to the database. A list shows all frames that will be updated as per the user data. Individual frames can be viewed which will compare the original frame data to the modified data. All changes between the original and modified frames will be highlighted to help the user more easily verify the import. The differences are calculated using a free library called google-diff-match-patch [8]. Highlighting is inferred from the text differences reported by the diff tool.

#### Commit to database

After the update is performed, the results of the update can be reviewed. This will provide a log of the successful and failed imports which can be used to verify the success of the import, or to track down problems with the data. Each individual import will be listed as either successful or failed, will be time stamped, and will refer to the original row of data in the spreadsheet which that update represents. Note that it may be possible to have several updates refer to the same row of data. At this point, the database is in a modified but unsaved state. If the user is satisfied with the update, the changes can be permanently saved to the database. Otherwise, the user can undo all changes to the database since the last save. The user will also be given the option of saving the change log to a file.

#### Import error detection

CycTools checks for errors and provides user feedback at several points during the import process. CycTools will directly reject syntax errors such as bad file formats of invalid references to database objects. Illegal database operations on the BioCyc database will cause failed imports in the final commit step, which will be flagged to users so that they can revert the database to an unmodified state. Imports with identifiers which cannot be resolved to existing database objects will be reported to the user as such.

Many errors in data entry are technically valid and thus cannot be differentiated from intentional input. If a slot label is misspelled, for example, CycTools will assume the user intends to create a slot using the misspelled label. The preview step provides users with a frame-by-frame comparison of the database in a modified and an unmodified state. Users are encouraged to browse the anticipated changes in order to detect any data entry errors that would otherwise be valid imports.

## Results and discussion

### Use case: MaizeCyc and CornCyc GO term annotation migration

MaizeCyc [9] and CornCyc [10] are two separate BioCyc databases both based on the *Zea mays* B73 RefGen_v2 gene models [11]. MaizeCyc is developed by Gramene [12] in collaboration with MaizeGDB [13] and CornCyc is developed by Plant Metabolic Network [14] and MaizeGDB [15-17]. Recent comparison between MaizeCyc and CornCyc revealed annotation differences in data content and quality despite both databases having been based on the same reference sequence [18]. MaizeCyc does not contain alternative splicing information; therefore each gene is only linked to a single gene product. CornCyc does contain alternative splicing information, where gene products linked to alternate splice variants are suffixed with a numerical identifier. It is interesting to note that even though MaizeCyc does not contain alternative splicing information, it still uses the numerical suffix convention for differentiating between alternately spliced proteins.

Recent curation efforts have provided GO term annotations for several proteins in the MaizeCyc database; however CornCyc version 4.0 does not currently contain any GO annotations. Since MaizeCyc and CornCyc both were created using the same sequence data and represent the same biology, the biological functions of MaizeCyc genes should be identical to those of CornCyc genes. In an effort to update the GO term annotations of the maize genome databases and ensure consistency across both databases, the manually curated GO annotations needed to be transferred from MaizeCyc to CornCyc.

All GO term assignments and their annotations were exported from MaizeCyc using a query to the Pathway Tools API and are provided in Additional file 4. GO term/Annotation pairs with an evidence code beginning with EV-EXP (i.e. experimentally verified annotations) were retained, while all others were removed. This represents the GO term annotations which have been manually verified by curators. Source protein objects were identified by their gene model name (e.g. GRMZM2G136161_P01) with the splice variant suffix attached (i.e. the _P01). This identifier was chosen as it is provided as a synonym in both MaizeCyc and CornCyc, which allows for accurate mapping between objects in both databases. Although MaizeCyc and CornCyc were built using the same gene model set, the internal frame IDs of the protein objects in Pathway Tools were generated with different syntax rules (i.e. most proteins in MaizeCyc begin with GBWI, while the equivalent proteins in CornCyc begin with GDQC).

**Figure 5:**
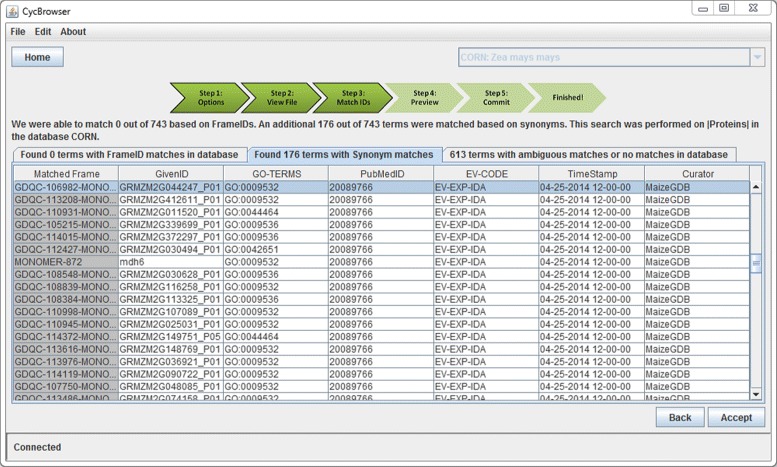
**GO term annotation import into CornCyc.** GO term annotations obtained from MaizeCyc are imported into CornCyc. User provided gene model identifiers are resolved to database frame IDs before import.

In order to ensure the most faithful mapping between MaizeCyc and CornCyc proteins, protein identifiers from MaizeCyc were used as query terms in a substring (synonym) search in CornCyc. Exactly matching splice variants provided 179 matches between MaizeCyc and CornCyc as seen in Figure 5. While an additional 5 matches can be made between this group of MaizeCyc and CornCyc proteins by relaxing the requirements to allow matches between alternate splice variants, these additional matches were not included in the final import. The remaining 458 gene products from MaizeCyc with EV-EXP annotations do not exist in CornCyc. The annotation data for the 179 matching protein GO term annotations were inserted into the CornCyc database using the CycTools import feature.

### Use case: creating strain-specific EcoCyc databases

Metabolic engineering projects lead to the generation of genetically unique strains. These altered strains are metabolically similar to the parent strain, but include a small number of modifications such as gene additions, deletions, or regulatory changes. Many novel strains may be created as a result of iterative engineering interventions performed on a parent strain. One possible solution to storing this information is to generate a new BioCyc database that is synchronized to the altered metabolism of the engineered strain. By using the most up-to-date version of EcoCyc and modifying it with information on engineering interventions, a new database is created which more accurately represents the engineered strain. This use case focuses on modifications to the *E. coli* organism performed for the increase of fatty acid production.

#### *E. coli* strains

Of the many strains of *E. coli* that are represented as model organism databases in the BioCyc database collection, EcoCyc has received the most manual curation. It is therefore desirable to leverage annotations from EcoCyc whenever possible while developing new strain databases. The metabolically engineered strains for which strain specific databases were developed in this study, strain ML103 and strain MLC115-1, were described in Liam et al. [19]. The genotype of ML103 is MG1655 *Δ*fadD. The genotype of MLC115-1 is MG1655 *Δ*fadD, *Δ*poxB, ackA-pta::cmR.

New regulatory links were predicted using the GTRNetwork software [20]. These results were derived for the MG1655 network, and so were applied to a copy of the wildtype EcoCyc database rather than the ML103 or MLC115-1 databases.

#### Copy EcoCyc

It is important to retain as much known information from the parent strain as possible, therefore the first step is to create a clone of the database representing the parent strain. Once the copy has been prepared, further modifications are necessary to align it to the altered metabolism of the engineered strain. In this case, the EcoCyc *E. coli* MG1655 database is downloaded (available free to academic users, requires registration) [21] and a copy is made to represent our strain specific database.

#### Strain specific updates to EcoCyc

Three types of data were added to the base EcoCyc database in order to represent changes in the engineered strain’s metabolism. A gene deletion in the strain is represented in EcoCyc by a deletion of the associated gene object and the gene object’s functionality. If the gene product is an enzyme, then that protein product is deleted and any reactions it catalyzes have that enzyme association removed from them. If the reaction has no existing enzymes which can catalyze the reaction, then the reaction is also removed. If the gene is a transcription factor, than the transcription factor is removed as well as any regulation objects in which that transcription factor was either a regulator or target. Preprocessing for this database modification simply requires compiling the list of genes to delete. CycTools automatically removes additional objects which are connected to the deleted gene as described above.

A thioesterase with altered specificity added to the strain improves specificity for specific fatty acid chain lengths. This does not represent novel metabolic functionality in the strain, but rather changes relative activities of an existing functionality. Since kinetic information and relative specificities of enzymes is not stored explicitly in current PGDBs, this information is best added to the comments section of the existing enzyme. Preprocessing in this case requires the user to explicitly write out the comment and provide the identifier of the enzyme to be modified.

The final modification made to the base EcoCyc database is the inclusion of novel computationally predicted transcription factor regulation. These regulatory interactions were predicted using GTRNetwork [20] and can be found in Additional file 5. Transcription factor regulatory interactions in EcoCyc are typically described by a regulation object which describes a transcription factor’s regulatory activity of a transcription factor binding site, but can also be described as a direct interaction between the regulating entity and the regulated gene. As the results produced in this computation prediction tool do not provide predicted binding sites, binding site information is not available for import. Preprocessing in this case requires the user to assemble the list of regulator and target interactions.

Each type of modification to the EcoCyc database must be made separately. In this case, the three modifications, gene deletions, thioesterase comment, and predicted regulation, represent three types of modification. Gene deletions are removed from the database by selecting the frame deletion option and loading the list of genes to be deleted. CycTools automatically removes extended links to the provided genes, such as their products and reactions. The thioesterase comment is performed as an update to an existing frame. A file with the comments is loaded and CycTools appends the new comment to the end of any existing comments on the enzyme. Importing novel predicted transcription factor regulation requires creating new regulation frames. This process is performed as two steps internally to CycTools. First, new frames are created using the user provided unique Frame IDs. An import step is then used to load the regulation data into the newly created regulation frames.

## Conclusions

Managing and migrating manual annotations in model organism databases are essential to maintaining high-quality biological data. In this work we present a software tool which provides a simple pipeline for the maintenance and transfer of manual annotations within and between BioCyc databases. CycTools improves user control over the import process by providing users with methods to edit slot values or slot-value annotations for any frame in a BioCyc database. CycTools also provides methods which allow users to create transcriptional regulatory frames or to delete frames through the import process.

CycTools provides methods that can make small or large-scale edits to a BioCyc database. Databases using the BioCyc framework typically contain between a few frames and several thousand frames. CycTools is capable of processing and displaying several thousand entries, but is limited to a single object type for each import. This means that CycTools is best suited to making many changes to a BioCyc database of a specific type, rather than making many small changes to various object types.

Tracking the changes made to a BioCyc database is made easier with CycTools. The BioCyc framework provides methods to credit an author or organization for frame edits. CycTools allows users to provide curator information which is stored in the BioCyc framework during the import process. CycTools also provides a change log of actions taken during import in order to assist users in recording changes and identifying problems.

In this manuscript, we have demonstrated the utility of CycTools by transferring GO annotations between two databases representing identical biology but having differing data content. We have also demonstrated the ability of CycTools to make several small scale changes to a database in order to customize the content to represent a non-model organism.

## Availability and requirements

**Project name:** CycTools**Project home page:**https://github.com/jrwalsh/CycTools/**Operating system(s):** Any platform supporting Java**Programming language:** Java**Other requirements:** Java 1.7+, Pathway Tools, JavaCycO

Pathway Tools must be installed and running on a Unix-like server system (due to use of the UnixDomainSocket class) and have the relevant PGDB installed. JavaCycO must be running in server mode on the same server as Pathway Tools. For remote connections, JavacycServer listens over a port connection, so this user selected port must be open to outside traffic. CycTools is written in Java and is thus cross-platform compatible, however Java must be installed on the client machine. The version of CycTools used in this manuscript can be found in Additional file 6.**License:** GNU GPL**Any restrictions to use by non-academics:** None

## Additional files

Additional file 1
**Examples of file formats.** This file contains examples of the “Lisp-format” and the “spreadsheet format” file formats accepted by the Pathway Tools import utility.

Additional file 2
**CycTools user’s manual and installation instructions.** This file contains instructions for installing CycTools and its dependencies. This file also includes descriptions of the options available in CycTools.

Additional file 3
**BioCyc database copy utility.** This file contains a bash shell script which will copy a BioCyc database file and automatically perform the necessary renaming steps required to differentiate the new copy from the old. This is required to allow both copies to be loaded in Pathway Tools memory simultaneously.

Additional file 4
**GO term annotation data from MaizeCyc.** This file contains the GO term annotations that were extracted from MaizeCyc and were imported into CornCyc. The file is formatted as comma delimited values.

Additional file 5
**Predicted regulatory links for EcoCyc.** This file contains the transcriptional regulatory links predicted by the program GTRNetwork for the *E. coli* network. These links include a regulatory mode such that “-” implies downregulation and “+” implies upregulation. An empty value in this column implies that the regulatory mode could not be determined.

Additional file 6
**CycTools source code.** This file contains the source code for the CycTools program.
