# Supplementary material for: A computational platform to maintain and migrate manual functional annotations for BioCyc databases
Source: BMC Syst Biol. 2014 Oct 12;8:115. doi: 10.1186/s12918-014-0115-1 (PMC4203924; doi:10.1186/s12918-014-0115-1)
Supplement: Additional file 2 — CycTools user’s manual and installation instructions. This file contains instructions for installing CycTools and its dependencies. This file also includes descriptions of the options available in CycTools. [file 12918_2014_115_MOESM2_ESM.zip › CycTools_User_Guide.pdf]

# Table of Contents

1 Introduction ........ 1
2 Installation Guide ........ 1
2.1 Install Java JDK ........ 1
2.2 Installing Pathway Tools ........ 2
2.3 Installing JavaCycO ........ 2
2.4 Installing CycTools ........ 2
3 CycTools Instructions ........ 3
3.1 Frame Viewer ........ 3
3.2 BioCyc Import ........ 4
3.2.1 Select Database and Import Type ........ 4
3.2.2 File Preview ........ 6
3.2.3 Preview Changes ........ 6
3.2.4 Commit to Database ........ 6
4 Using iPlant Atmosphere Virtual Machines ........ 6
4.1 Running a Server on an Atmosphere Virtual Machine ........ 6
4.2 Launching an Atmosphere Virtual Machine ........ 7

# 1 Introduction

CycTools is an interface for accessing and updating data in a BioCyc family Pathway/Genome Database (PGDB). Access to the database provided by the Pathway Tools programmatic API. The JavaCycO library allows conversion of Java based commands into queries which can be understood by the Pathway Tools API. In order to use CycTools, a server running Pathway Tools and JavaCycO must be available. Because of the requirements of JavaCycO, this server must be running on a Unix-like environment, such as Ubuntu. JavaCycO requires the Java Runtime Environment (JRE) version 1.7 or higher on both the host and client machines. Because the server portion of this tool must be run on a linux-like OS, the installation guide is provided for Linux (Ubuntu) only. The client portion of this tool can be run on any OS that supports java 1.7 or above.

# 2 Installation Guide

While CycTools does not need to be installed to run, it depends on other software which must be installed before CycTools can be used. The general steps to using CycTools include:

1. Install Java SDK on Server
2. Install Pathway Tools on Server
3. Install JavaCycO on Server

After installation is complete, running CycTools will involve launching Pathway Tools in API mode, launching JavaCycO, then launching CycTools and connecting to the JavaCycO instance. The following instructions assume the machine is running Ubuntu 12.04.

## 2.1 Install Java JDK

Java 1.7 JDK or higher must be installed on both the host and client machine. For windows or Mac machines, follow the instructions here http://www.java.com/en/download/manual.jsp.

1) Check java version:
   - java -version

   Verify installation of version 1.7 or above of the JDK (java development kit). The JRE (Java Runtime Environment) will not have the tools necessary to compile JavaCycO.
2) Install java jdk
   - sudo apt-get install openjdk-7-jdk
3) Verify that the correct version of java is set as default
   - sudo update-alternatives --config java

   If only one java version is installed, no action is needed. If multiple versions are installed, select the java-7 jdk option (see figure 1).

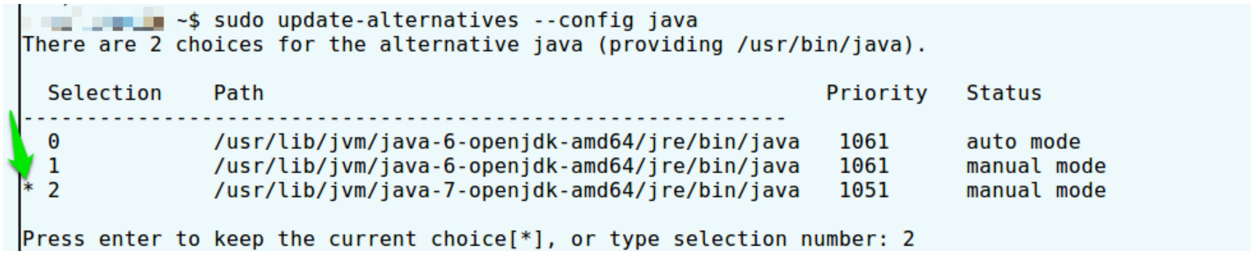


*Figure 1: Selecting Java version from installed versions.*

## 2.2 Installing Pathway Tools

A local installation of Pathway Tools on a Unix-like system is required for accessing PGDB information through CycTools. The server that Pathway Tools is installed to can be the same machine that CycTools will be run from, or another machine accessible to the client. Installation instructions for Pathway Tools can be found on the pathway tools website. Note that while Pathway Tools is currently free for academic research purposes, a license request must be made to access the installer. At of the time of this writing, the request can be made here: http://biocyc.org/download-bundle.shtml. This guide assumes Pathway Tools version 17.5 is installed using the default settings and file locations as a single user (i.e. not as the Administrator).

## 2.3 Installing JavaCycO

JavaCycO relies on the libunixdomainsocket.so libraray, which uses a UnixDomainSocket to communicate with Pathway Tools. Since UnixDomainSocket is only available on Unix-like systems, JavaCycO must be installed on a Unix-like operating system. JavaCycO must be installed on the same machine as the Pathway Tools installation.

1. Copy the CycTools_v0.1.0-beta.jar file to your preferred installation directory. It is recommended to install JavaCycO in the “ptools-local” directory created by Pathway Tools.
2. Run the install_JavaCycO.sh file by double clicking the file and selecting “Run in Terminal”.
   - Install location defaults to current folder, but can be changed by user during install.
   - If you have java installed in a location other than the default for Ubuntu, you will need to modify the installer script in a text editor to point to the location of the jni.h file.

The server portion of JavaCycO, the JavacycServer, can now be run by double clicking the runJavacycServer.sh file. This must be run in terminal mode.

## 2.4 Installing CycTools

CycTools is a stand-alone java application which can run on any operating system supporting java. Java 1.7 must be installed on the client machine in order to run CycTools. On Linux-type systems, you may have to set the permissions to allow CycTools.jar file to be executable by using chmod u+x on the file. Run CycTools either by double clicking the jar directly (may not work if jar files are not associated with java on your machine), double clicking the appropriate run_CycTools file (bat file for windows, sh file for linux) or with the terminal command:

- java –jar CycTools.jar

In order to successfully connect to a JavaCycO instance, Pathway Tools must be running on a linux-like machine in API mode:

- ./pathway-tools –api
  * From the directory pathway tools was installed to in step 2.2

The JavaCycO server must also be running on the same machine as Pathway Tools:

- ./runJavacycServer.sh
  * From the directory JavaCycO was installed to in step 2.3

# 3 CycTools Instructions

CycTools can connect to a local or remote instance of JavaCycO. Enter the IP address of the server (localhost if on the same machine) and the port (default 4444). The username and password are not necessary unless set up during the JavaCycO installation.

## 3.1 Frame Viewer

The frame viewer tool is used to inspect the data contents of a single frame in the PGDB. Frame names can be entered directly, or a substring search can be performed to look for frames with a given substring in their name.

Start by entering a frame ID or search term in the search box. Select the frame type that you would like to search for (exact frame ID matches will be returned regardless of type selected). Then press submit (or press enter in the search box). If any matches are found, a window will pop-up displaying the search results. If an exact frame ID match was found, it will be indicated in the top of the display window. Other frames which match by substring will be displayed below. Options are displayed with the frame ID in parenthesis followed by the display name of the frame (if one exists). Selecting a frame and pressing OK will load that frame in the frame view area.

Frames are displayed in simple ASCII text. The structure of the display is as follows: the name of the frame is displayed first. This is followed by a series of slot labels, one per line, with the number of values in that slot in parenthesis behind the slot label. Slot values for a given slot name are shown, one per line, indented once from their slot label. Some slot values have slot-value-annotations. If this is the case, on the line following a slot value will be two dashes (--) followed by the annotation label, two indents in. Annotation values follow the annotation label.

After all slots, values, and annotations, the superclasses of the frame are printed, along with the JavaCycO object type that was associated with this frame and the database that the frame was loaded from.

The frame viewer can display any frame in the PGDB, including instance and class frames. Most slot and annotation labels exist as frames which can be viewed.

## 3.2 BioCyc Import

The BioCyc Import tool is designed to make importing spreadsheet data into frame objects in the PGDB easy. The import utility takes as input a spreadsheet formatted file of data, maps the data to frames in the PGDB, previews the resulting changes to the PGDB, and performs the update of the PGDB with the spreadsheet data.

### 3.2.1 Select Database and Import Type

Import types include

1. Slot Value Import
2. Annotation Value Import
3. GO Term Import
4. Create Transcriptional Regulation
5. Delete Frame and Dependents

For the slot value option, the first column must be the exact frame ID of the frame to be modified. The following column headers must match the slot labels of the frame to be changed. Values in that column represent slot values (and can be separated by the multiple value delimiter if multiple values are desired). As many columns as desired can be imported at once. Only one frame object is allowed per line, a single frame can have multiple rows of updates.

For the annotation value option, the first column must again be the exact frame ID of the frame to be modified. The second column must be the slot label of the slot to be annotated. The values in this column are the values to be annotated. *Note that you cannot use multiple values here, or it would not be possible to know what value is to be annotated!*

The GO Term option uses the format ID, GO Term, PubMedID, EVCode, Time Stamp (mm-dd-yyyy hh-mm-ss), Curator. This option will trigger Pathway Tools to automatically import additional GO Term information for any GO annotations imported. The time stamp will be converted to Lisp date time format before importing.

The Create Transcriptional Regulation option uses the format Regulator, Regulatee, Mode. It will create new regulation instances in setting the regulator as having a transcriptional regulatory effect on the regulate of the type specified in mode ("-" for downregulation, "+" for upregulation). The regulator and regulatee IDs must be valid internal identifiers in the database (alternate identifiers cannot be used). As such, the alternate identifier search feature is skipped when performing this type of import. The new regulation frames will be assigned unique sequential identifiers automatically.

The Delete Frame option only requires the ID of the frames to be deleted in the first column. This frame and all dependents will be deleted from the database. If the frames to be deleted are gene frames, then all protein products and associated enzyme reactions will be deleted.

### 3.2.2 Select Options

Select the data file which is to be uploaded to the PGDB.

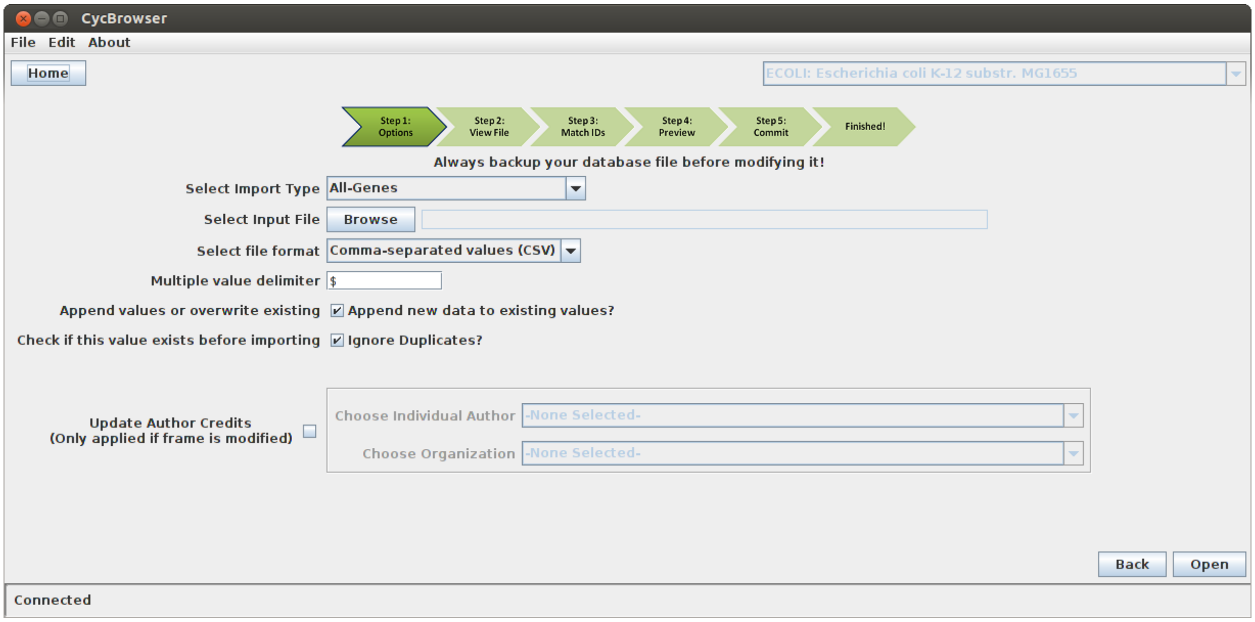


*Figure 2: CycTools Import Options.*

1) Select Import Type: when using the Slot Value, Annotation Value import, or Delete Frame import, this should by the frame type that is being updated. When using GO Term import, this should be set to Proteins (where GO annotations are stored). When using the Create Transcriptional Regulation import, this setting is ignored. This setting is used in the search feature to narrow search results when matching an alternate identifier to frames in the database.
2) Select Input File: select the data file to import
3) Select File Format: the spreadsheet can either be comma-separated values or tab-separated values
4) Multiple Value Delimiter: if multiple values are to be added to a single slot, they can be separated with the delimiter given here. The delimiter is interpreted literally (non-regex) and defaults to "$". If multiple value imports are not used, this is optional.
5) Append new data to existing values?: If selected, the new data imported will be added to the existing data in the PGDB. If not selected, the existing data will be replaced entirely with the new data. *Only slots explicitly being imported will be affected*
6) Ignore Duplicates: if selected, CycTools will first check to see if the exact value already is contained by the frame. If so, it will not be added again. Especially useful to check this option if running the same import file multiple times.
7) Update Author Credits: this option can be used to assign one individual and/or one organization to give curator credit to when importing changes. The individual or organization must already exist in the database, and will be added to the "Credits" slot as having "Revised" the frame. This should not be set when using the Delete Frame import.

### 3.2.3 File Preview

The second step is the File Preview step. The contents of the file will be loaded to the screen for review. Ensure that the file contains the correct information and that the file is being loaded correctly (comma/tab separated). Note that the multiple value delimiter does not affect preview at this stage. Pressing preview will go to the next screen. This step may take several minutes, as every frame being updated must first be downloaded.

### 3.2.4 Preview Changes

The third step is to preview the changes to the database. At this stage, no changes have been made to the data in the database. The list on the top shows all frames which are being updated as per the spreadsheet data. Selecting a frame will call up the original frame data in the bottom left text area, and the modified data in the bottom right text area. All changes will be highlighted in the text to assist in viewing the differences. Additionally, the next diff button can be used to scan through the updates made to the data step by step. Be sure to verify that the changes to the database are the changes intended. A checkbox is provided that will filter out any frames from the list that do not result in modified data after the import. This can be useful if the same import data is used multiple times. If the user is satisfied with the proposed changes, the update database button can be pressed, which will perform the import and modify the database. This step may take several minutes.

### 3.2.5 Commit to Database

After the update is performed, the results of the update can be reviewed in the final screen. This will provide a log of the successful and failed imports. Use this information to verify the success of the import, or to track down problem data. Each individual import will be listed as either success or fail, will be timestamped, and will refer to the original row of data in the spreadsheet which that update represents. Note that it may be possible to have several updates refer to the same row of data.

At this point, the database is in a modified but unsaved state. If the user is satisfied with the update, the save button will save the changes. Otherwise, the cancel button will undo all changes to the database. The user also has the option of saving the change log to file.

**Remember:**

1) Always save copy of database before making changes!
2) Make sure database is unmodified at start of the import process!
3) Do not have multiple users accessing the database at this time (either through CycTools or Pathway Tools)

# 4 Using iPlant Atmosphere Virtual Machines

## 4.1 Running a Server on an Atmosphere Virtual Machine

Atmosphere provides computing resources to academic users in the form of virtual machines which can be launched and administered by the user. This provides an ideal setting for testing CycTools without

having to set up a linux-based Pathway Tools installation on a local machine. Atmosphere virtual machines can be set up to minimize the effort needed to run user software

## 4.2 Launching an Atmosphere Virtual Machine

After creating an account at [http://www.iplantcollaborative.org/](http://www.iplantcollaborative.org/) and logging in to the Atmosphere service [https://atmo.iplantcollaborative.org/login/](https://atmo.iplantcollaborative.org/login/) , you can create a virtual machine on which to run CycTools. Be sure to launch atmosphere at [https://atmo.iplantcollaborative.org/application](https://atmo.iplantcollaborative.org/application).

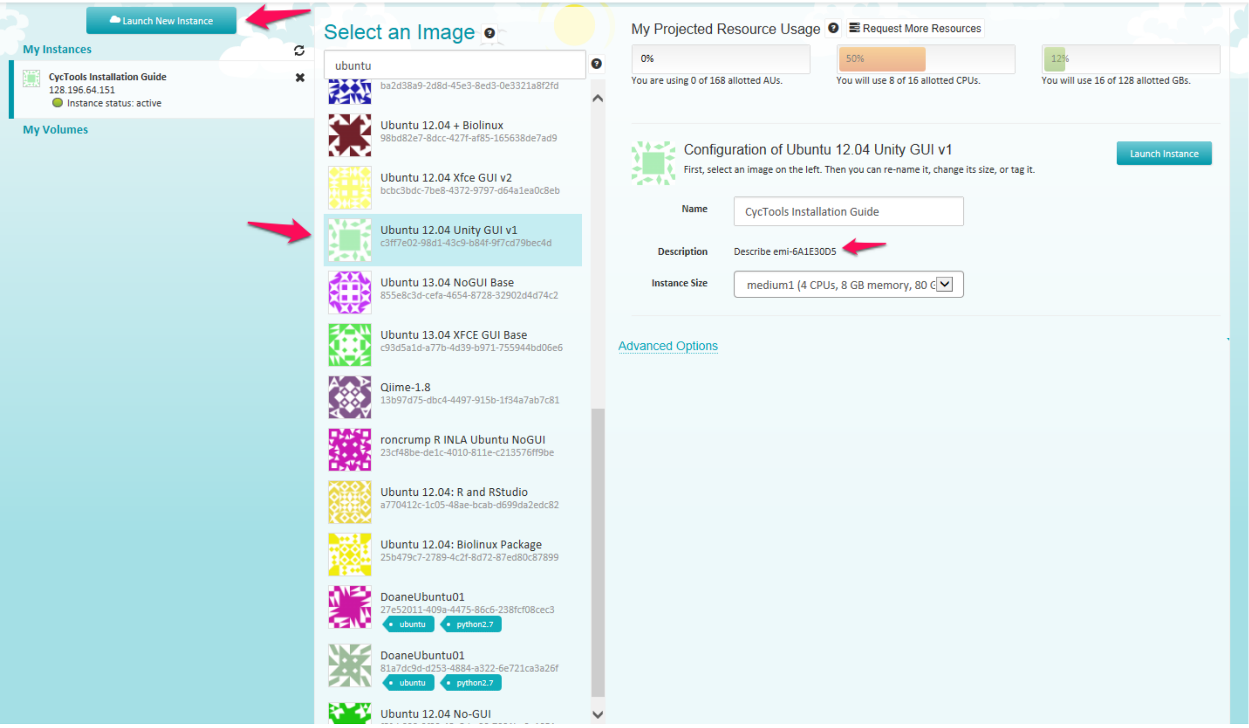


*Figure 3: Atmosphere home screen, launching a machine instance.*

Launch a new instance using any suitable linux-based operating system. (CycTools was tested using Ubuntu 12.04 Unity GUI v1 for this guide). Once the machine has launched, access to the machine can be gained through several methods, including the web shell, VNC (for supported virtual machines), or through a ssh or terminal program such as putty (windows).

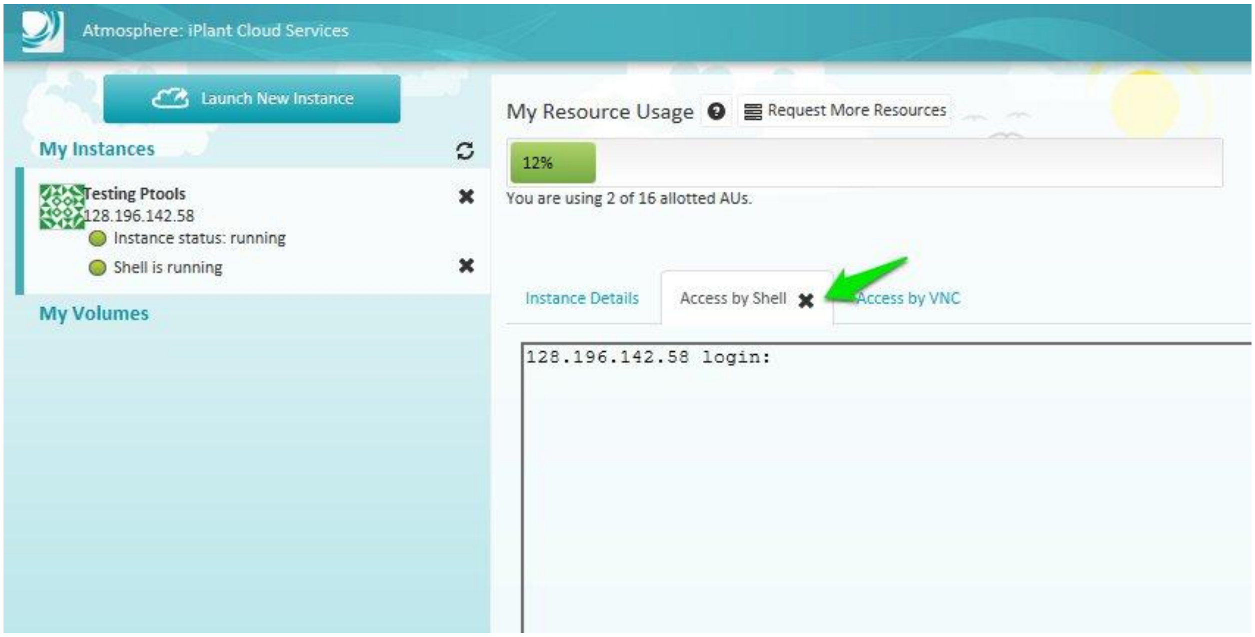


*Figure 4: Web based shell access.*

Log into the machine using your atmosphere username and password. You will need terminal (command line) access to the machine to continue.

Connecting through a terminal of your local machine, use the following command, replacing with your username and your virtual machines IP address:

*ssh -X username@123.456.789.10*
